# Tissue plasminogen activator inhibits NMDA-receptor-mediated increases in calcium levels in cultured hippocampal neurons

**DOI:** 10.3389/fncel.2015.00404

**Published:** 2015-10-09

**Authors:** Samuel D. Robinson, Tet Woo Lee, David L. Christie, Nigel P. Birch

**Affiliations:** ^1^School of Biological Sciences and Centre for Brain Research, University of AucklandAuckland, New Zealand; ^2^Brain Research New Zealand, Rangahau Roro Aotearoa, University of AucklandAuckland, New Zealand

**Keywords:** tissue plasminogen activator, NMDA receptor, calcium signaling, hippocampal neurons, synapse

## Abstract

NMDA receptors (NMDARs) play a critical role in neurotransmission, acting as essential mediators of many forms of synaptic plasticity, and also modulating aspects of development, synaptic transmission and cell death. NMDAR-induced responses are dependent on a range of factors including subunit composition and receptor location. Tissue-type plasminogen activator (tPA) is a serine protease that has been reported to interact with NMDARs and modulate NMDAR activity. In this study we report that tPA inhibits NMDAR-mediated changes in intracellular calcium levels in cultures of primary hippocampal neurons stimulated by low (5 μM) but not high (50 μM) concentrations of NMDA. tPA also inhibited changes in calcium levels stimulated by presynaptic release of glutamate following treatment with bicucculine/4-aminopyridine (4-AP). Inhibition was dependent on the proteolytic activity of tPA but was unaffected by α_2_-antiplasmin, an inhibitor of the tPA substrate plasmin, and receptor-associated protein (RAP), a pan-ligand blocker of the low-density lipoprotein receptor, two proteins previously reported to modulate NMDAR activity. These findings suggest that tPA can modulate changes in intracellular calcium levels in a subset of NMDARs expressed in cultured embryonic hippocampal neurons through a mechanism that involves the proteolytic activity of tPA and synaptic NMDARs.

## Introduction

NMDA receptors (NMDARs) play an essential role in the regulation of synaptic strength in the brain (Lau and Zukin, 2007; Traynelis et al., 2010; Paoletti, 2011). These gated cation channels are activated by the excitatory neurotransmitter glutamate and are essential mediators of brain plasticity, impacting synaptic structure and function. NMDA receptor activation leads to rapid alterations in synaptic strength that contribute to long-term potentiation (LTP) and long-term depression as well as longer term changes that are important for maintaining neuronal network function. Stimulation of NMDARs leads to activation of calcium-dependent signaling pathways and changes in expression of plasticity-related genes. It is becoming increasingly clear that these signaling properties are dependent on receptor localization and subunit composition. NMDARs are mobile and move laterally between synaptic and extrasynaptic pools (Lau and Zukin, 2007; Bard and Groc, 2011). Synaptic and extrasynaptic receptors can stimulate different signaling pathways resulting in different neuronal responses. These may be mediated by differences in NMDA receptor subunit composition at the different sites, enabling synaptic and extrasynaptic receptors to associate with different signaling molecules (Rao and Finkbeiner, 2007). NMDARs form tetrameric complexes. The subunit composition of these complexes is diverse and plastic, resulting in a large number of receptor subtypes that varies during development and at adult synapses (Paoletti, 2011).

Tissue-type plasminogen activator (tPA) is a member of the serine protease family most well known for its role in vascular thrombolysis where it activates the zymogen plasminogen to form plasmin, which degrades fibrin and remove blood clots (Cesarman-Maus and Hajjar, 2005). tPA has been identified in both the developing and adult nervous system where a number of distinct roles have been proposed (Sappino et al., 1993; Friedman and Seeds, 1994; Ware et al., 1995; Teesalu et al., 2004). tPA is released from neurons following membrane depolarization (Lochner et al., 2006) and regulates LTP and synaptic plasticity. A genetic deficiency of tPA or inhibition of tPA activity leads to a loss of LTP (Frey et al., 1996; Huang et al., 1996; Calabresi et al., 2000) while overexpression of tPA or addition of recombinant tPA leads to prolonged LTP (Baranes et al., 1998; Madani et al., 1999). These and other observations are consistent with roles for tPA in learning and memory (Centonze et al., 2002; Pawlak and Strickland, 2002; Fernández-Monreal et al., 2004b; Benchenane et al., 2007). Several mechanisms may underpin these effects. At the cellular level tPA has been linked to the formation of perforated synapses (Neuhoff et al., 1999) and presynaptic varicosities (Baranes et al., 1998) as well as changes in dendritic spines (Mataga et al., 2004; Pawlak et al., 2005). Mechanistically, the proposed roles for tPA in synaptic plasticity mainly focus on changes in the proteolytic microenvironment impacting the remodeling of extracellular matrix (Wu et al., 2000; Bukhari et al., 2011) and synaptic connectivity, including cleavage of neurotrophins (Pang et al., 2004; Barnes and Thomas, 2008) and neurotransmitter receptors (Samson et al., 2008a; Macrez et al., 2010; Ng et al., 2012).

In this study we have investigated the effects of tPA on NMDA-mediated changes in intracellular calcium levels using a primary embryonic rat hippocampal culture model (Banker and Cowan, 1977). Calcium flux was stimulated directly with varying concentrations of the glutamate receptor agonist NMDA. We also stimulated presynaptic release of glutamate using a γ-aminobutyric acid A (GABA_A_) receptor antagonist and potassium channel blocker, 4-aminopyridine (4-AP; Hardingham et al., 2002). Possible roles for plasmin and low density lipoprotein receptor-related protein 1 (LRP-1), an endocytic and signaling receptor that interacts with tPA (Zhuo et al., 2000) were also examined.

## Materials and Methods

### Materials

Recombinant human tPA (Actilyse^®^) was a generous gift from Boehringer Ingelheim (Auckland, New Zealand) or purchased from Biopur (Reinach, Switzerland). For some experiments the excipients in Actilyse^®^ tPA were removed by dialysis against HEPES (Samson et al., 2008b). NMDA, MK-801 and amino-5-phosphonovalerate (APV) were purchased from Sigma Aldrich (Auckland, New Zealand). Nimodipine, bicuculline and 4-AP were purchased from Tocris (MO, USA). 2,7-Bis-(4-aminobenzylidene)-cycloheptan-1-one dihydrochloride (tPA-STOP) was purchased from American Diagnostica (Greenwich, CT, USA). Human α_2_-antiplasmin was purchased from MyBioSource (San Diego, CA, USA). Receptor-associated protein (RAP) was produced as a glutathione S-transferase fusion protein and purified by glutathione-affinity chromatography. The glutathione S-transferase tag was removed by thrombin cleavage prior to use. The active site mutant human tPA (S478A) was purchased from Molecular Innovations (MI, USA).

### Primary Cell Culture

The use of animals in this research was approved by the University of Auckland Animal Ethics Committee. Primary hippocampal cultures were prepared from embryonic (E18) Wistar rats as described previously (Borges et al., 2010; Lee et al., 2015). Briefly, dissected hippocampi were dissociated using papain and plated at 20,000 cells/well in clear-bottom, black-walled 96-well amine plates (BD Biosciences, Auckland, New Zealand). Cultures were maintained for 14–17 days *in vitro*, in Neurobasal medium containing 1 × B27 and 1 × Glutamax (all from Invitrogen, Carlsbad, CA, USA), with half medium changes 24 h following plating and then at 7 day intervals.

### Calcium Assays

All experiments were performed on cultures maintained for 14–17 days *in vitro*. Cells were loaded with Fluo-4 AM (Life Technologies) according to the manufacturer’s instructions. Calcium responses were recorded on an Envision plate reader (Perkin Elmer, MA, USA) using the following settings: excitation filter, FITC 485 nm; emission filter, 520 nm. Each well was recorded individually, with 15 s of baseline recording, followed by injection of agonist and recording of the response for a further 45 s. Antagonists were added manually 5 min (tPA) or 15 min (MK-801, APV, nimodipine, tPA-STOP, α_2_-antiplasmin, RAP), prior to recording. Raw fluorescence data were converted to ΔF/F_0_; where F_0_ is the average fluorescence over the first 15 s of recording prior to addition of agonist (baseline) and ΔF is F_max_−F_0_.

### Statistics

The area under the curve (AUC) from 0 to 45 s was determined and used as the dependent variable for all statistical analyses. These data were first transformed using a square root transform to ensure that there was homogeneity of variance and an approximately normal distribution of residuals in the fitted statistical models. The data for all NMDA treatments were analyzed as a complete set. This data set contained 24 individual plates of cells, 121 treatment by plate biological replicates and 351 individual data points. The data for the bicuculline and 4-AP treatments were analyzed separately, consisting of 4 plates of cells, 19 treatment by plate replicates and 53 individual data points. Statistical analysis was conducted in R 3.0.2 (R Development Core Team, 2013) using linear mixed models of the transformed data. Data were modeled with a one-factor randomized block design containing a fixed effect for treatment and each plate considered a block. This ensured that each experimental unit (block) was a replicate plate of cells, with the technical replicates within a plate functioning as sampling units. As the data contained a nested structure (technical replicates nested within biological replicates) and were not balanced (not all treatments were in all plates, differing number of technical replicates), the mixed models were fitted by restricted maximum likelihood (REML) instead of general linear model/ANOVA (Littell et al., 2002; Lazic, 2010). The statistical model equation (Littell et al., 2002) was *y_ijk_* = *μ* + *α_i_* + *b_j_* + *(αb)_ij_* + *e_ijk_* where *y_ijk_* is the transformed AUC measurement for the *k*th technical replicate for treatment *i* in plate *j*, μ + *α_i_* is the mean transformed AUC measurement for treatment *i*, *b_j_* is the random effect associated with plate *j*, *(αb)_ij_* is the plate by treatment random effect (biological replicates) and *e_ijk_* is the random error associated with technical replicate *k* for treatment *i* in plate *j*. The R command *lmer* from the package *lme4* (Bates et al., 2013) was used to fit the linear mixed models using the following model specification: *transformed*_*AUC*~ *treatment* + (1|*plate*_*id*) + (1|*plate*_*id*:*treatment*). All statistical tests were planned comparisons between a treatment and the appropriate control and were calculated as contrasts from the fitted models with* p*-values obtained using degrees of freedom determined by the Kenward-Roger method using packages *lsmeans* (Lenth, 2013) and *pbkrtest* (Højsgaard, 2014). As these were decided *a priori* no correction for multiple tests was applied (Ruxton and Beauchamp, 2008). Data are plotted as backtransformed means and SEM.

## Results

### tPA Inhibits the Calcium Response of Hippocampal Neurons Activated with Low but not High Concentrations of NMDA

**Figure 1:**
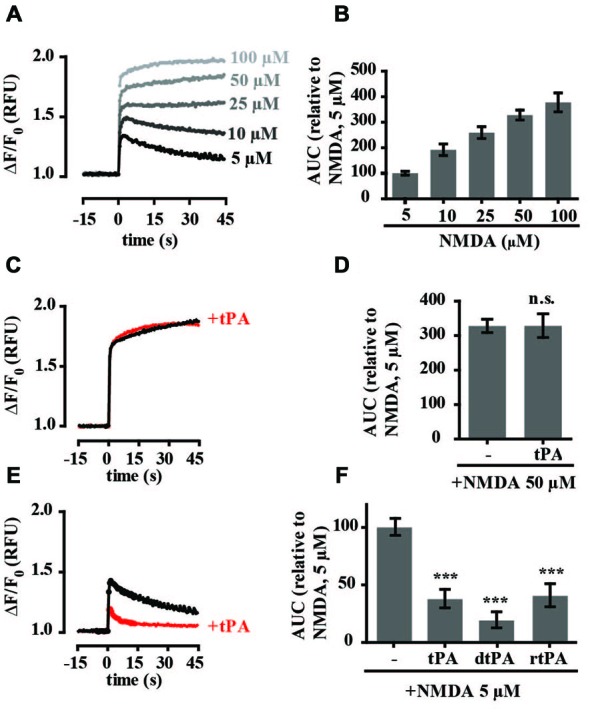
**Tissue-type plasminogen activator (tPA) inhibits increases in intracellular calcium in cultured rat hippocampal neurons stimulated with low (5 μM) but not high (50 μM) concentrations of NMDA. (A)** Baseline Fluo-4 fluorescence was monitored in hippocampal cultures for 15 s prior to the addition of NMDA, at time = 0, to final concentrations ranging between 5 and 100 μM. Fluo-4 fluorescence was monitored for a further 45 s. Raw fluorescence values were converted to ΔF/F_0_, where F_0_ is the average fluorescence over the first 15 s of recording prior to addition of agonist (baseline) and ΔF is F_max_−F_0_. **(B)** The responses in A were quantitated by measuring the area under the curve (AUC) and are presented relative to the AUC for 5 μM NMDA (100%). Results are from three independent experiments. Error bar, SEM. **(C,D)** Hippocampal cultures were preincubated with tPA (40 μg/ml) for 5 min and Fluo-4 fluorescence monitored before and after addition of NMDA to a final concentration of 50 μM. Data was collected as in A and quantitated as in B. n.s, not significant. **(E)** Hippocampal cultures were preincubated with tPA (40 μg/ml) for 5 min and Fluo-4 fluorescence monitored before and after addition of NMDA to a final concentration of 5 μM. **(F)** Quantitation of responses to tPA (Actilyse, sourced from Boehringer Ingelheim), rtPA (rtPA sourced from Biopur) and dtPA (dialysed Actilyse). RFU, Relative Fluorescent Units, ***p < 0.001. Error bar, SEM.

To study the effect of recombinant tPA on NMDA-mediated calcium flux, intracellular calcium levels were monitored in embryonic hippocampal neurons cultured between 14 and 17 DIV (days *in vitro*), using a Fluo-4-based calcium assay and a high speed fluorometric plate reader. Treatment with the NMDAR agonist NMDA alone (5–100 μM) resulted in a rapid and concentration-dependent increase in intracellular calcium levels (Figures 1A,B). The fluorescent profiles indicated differences in handling between lower (<10 μM) and higher (>10 μM) concentrations of NMDA. Both responses were characterized by a rapid influx of calcium (the amplitude of which was concentration-dependent) which in the former gradually returned towards baseline, but in the latter plateaued, resulting in sustained calcium levels. In some instances small oscillations were observed at the lower NMDA concentrations. To investigate the effect of tPA on the calcium response, Fluo-4-loaded neurons were treated with tPA. Addition of tPA alone (40 μg/ml) did not produce any detectable change in Fluo-4 fluorescence (Supplementary Figure 1) and the calcium response to 50 μM NMDA was unaffected by pre-incubation with tPA (Figures 1C,D). However, the calcium response to 5 μM NMDA was significantly reduced to 38 ± 8% of control (Figures 1E,F). The tPA used in these experiments (Actilyse^®^) contains a number of excipients, including L-arginine, which has been suggested to be toxic to neurons (Oh et al., 2005; Samson et al., 2008b). To examine for any effects of the excipients in the Actilyse^®^ tPA on the calcium response we repeated the experiments with a dialysed preparation of Actilyse^®^ tPA (dtPA) and a second commercial recombinant tPA, Biopur tPA, which does not contain excipients. Both produced similar inhibition of the 5 μM NMDA-mediated calcium response (Figure 1F), supporting the designation of tPA as the active agent. For all subsequent experiments Actilyse^®^ tPA, which will be referred to from now simply as tPA, was used.

### tPA Modulates Trans-Synaptic Stimulation of NMDA Receptors

**Figure 2:**
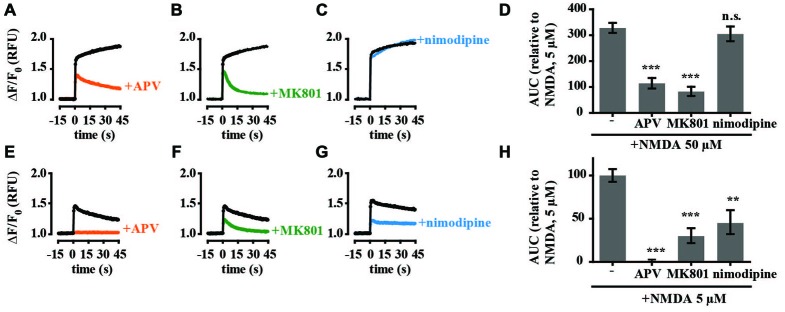
**Calcium responses of cultured hippocampal neurons to high (50 μM) and low (5 μM) concentrations of NMDA are pharmacologically distinct.** Hippocampal cultures were preincubated with antagonists for 15 min before recording calcium responses. Baseline Fluo-4 fluorescence was monitored for 15 s prior to the addition of NMDA (**A–C**, 50 μM NMDA; **E–G**, 5 μM NMDA) at time = 0, then monitored for a further 45 s. Raw fluorescence values were converted to ΔF/F_0_, where F_0_ is the average fluorescence over the first 15 s of recording prior to addition of agonist (baseline) and ΔF is F_max_−F_0_. Antagonists tested were amino-5-phosphonovalerate (APV) (**A,E**; 50 μM), MK-801 (**B,F**; 10 μM); nimodipine (**C,G**; 10 μM). **(D,H)** Responses were quantitated by measuring the AUC and are presented relative to the AUC for 5 μM NMDA (100%). RFU, Relative Fluorescent Units; **p < 0.005, ***p < 0.001. Error bar, SEM.

**Figure 3:**
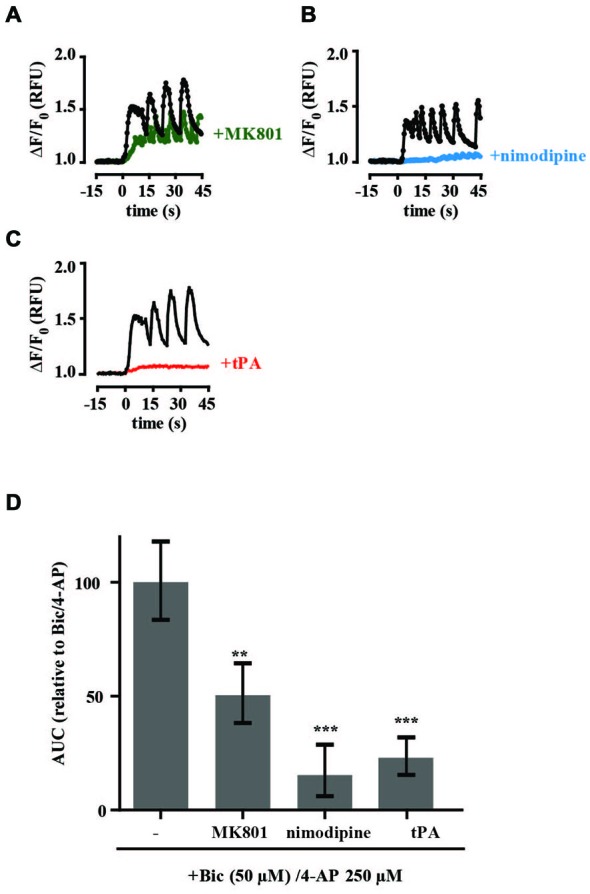
**tPA inhibits the calcium response of cultured hippocampal neurons stimulated by presynaptic release of glutamate.** Hippocampal cultures were preincubated with antagonists (15 min) or tPA (5 min). Baseline Fluo-4 fluorescence was monitored for 15 s prior to the addition of bicucculine (50 μM) and 4-aminopyridine (4-AP, 250 μM) at time = 0. Fluorescence was monitored for a further 45 s and values were converted to ΔF/F_0_, where F_0_ is the average fluorescence over the first 15 s of baseline recording. Agents tested were **(A,B)** nimodipine (10 μM), **(C)** tPA (40 μg/ml). **(D)** Responses were quantitated by measuring the AUC and are presented relative to the AUC for 5 μM NMDA (100%). RFU, Relative Fluorescent Units; **p < 0.005, ***p < 0.001. Error bar, SEM.

To investigate the NMDAR responses in more detail we assessed the effects of glutamate and calcium channel antagonists on tPA-sensitive calcium flux. The selective competitive NMDAR antagonist APV (50 μM) and the use-dependent NMDAR open channel blocker MK-801 (10 μM) both significantly inhibited the calcium response of neurons treated with 50 μM NMDA while the L-type voltage-gated calcium channel blocker nimodipine (10 μM) had no significant effect on calcium levels (Figures 2A–D). The calcium response of hippocampal neurons to 5 μM NMDA was also blocked by APV and MK-801. However, in contrast to stimulation with 50 μM NMDA, nimodipine also inhibited the response (Figures 2E–H). These results are consistent with previously published data (Jensen and Wang, 1996; Soriano et al., 2006) and support trans-synaptic activation of the postsynaptic cell by glutamate at 5 μM NMDA, and direct stimulation of postsynaptic NMDARs at 50 μM NMDA. We further investigated an effect of tPA on the calcium response stimulated by presynaptic release of glutamate using the GABA_A_ receptor antagonist bicuculline (50 μM) and the potassium channel blocker 4-AP (250 μM; Hardingham et al., 2001b). Treatment resulted in synchronous spontaneous calcium oscillations suggestive of synaptic coupling between neurons (Figures 3A–C). These oscillations were markedly reduced by MK-801 and nimodipine (Figures 3A–C,D). Importantly, the calcium oscillations were also markedly inhibited by tPA (Figure 3D).

### The Proteolytic Activity of tPA is Required for its Inhibitory Effect on NMDA-Mediated Changes in Intracellular Calcium Levels

**Figure 4:**
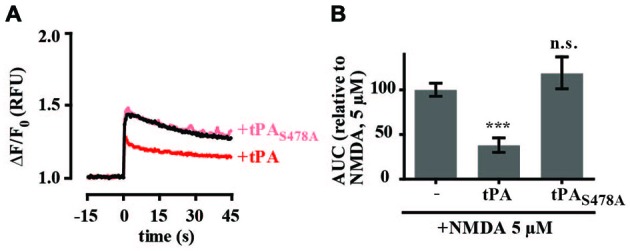
**The proteolytic activity of tPA is required for inhibition of NMDA-induced calcium influx. (A)** Hippocampal cultures were preincubated with tPA or the enzymatically inactive tPA mutant tPA_S478A_ (40 μg/ml) for 5 min. Baseline Fluo-4 fluorescence was monitored for 15 s prior to the addition of NMDA (5 μM), at time = 0. Fluo-4 fluorescence was monitored for a further 45 s. Raw fluorescence values were converted to ΔF/F_0_, where F_0_ is the average fluorescence over the first 15 s of recording prior to addition of agonist (baseline) and ΔF is F_max_−F_0_. **(B)** The responses in A were quantitated by measuring the AUC and are presented relative to the AUC for 5 μM NMDA (100%). RFU, Relative Fluorescent Units; n.s, not significant; ***p < 0.001. Error bar, SEM.

As proteolytic and non-proteolytic mechanisms have been reported to modulate NMDA-mediated calcium levels, we tested the effects of tPA-STOP, a reversible competitive inhibitor of trypsin-like serine proteases, as well as an enzymatically inactive tPA mutant. Unexpectedly, preincubation of cultures with 1 μM tPA-STOP alone almost completely inhibited intracellular calcium changes activated by 5 μM NMDA (Supplementary Figure 2). This result suggested an off-target effect of tPA-STOP and so it was not used in any further experiments. In a second approach we tested the effect of an enzymatically-inactive tPA mutant, with the active site Ser_478_ residue mutated to alanine. In contrast to tPA, the enzymatically-inactive tPA_S478A_ had no effect on 5 μM NMDA-mediated changes in calcium levels (Figures 4A,B).

### Inhibition of NMDA-Induced Calcium Influx by tPA is Independent of Plasmin and LRP-1

**Figure 5:**
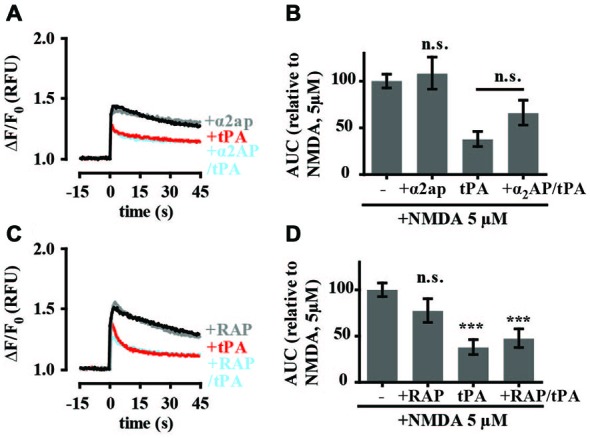
**tPA inhibition of NMDA-induced calcium influx is independent of plasmin and lipoprotein receptor-related protein 1 (LRP-1). (A)** Hippocampal cultures were preincubated with tPA (40 μg/ml; for 5 min) or α_2_-antiplasmin (140 nM; for 15 min) and tPA (for 5 min). Baseline Fluo-4 fluorescence was monitored for 15 s prior to the addition of NMDA (5 μM), at time = 0. Fluo-4 fluorescence was monitored for a further 45 s. Raw fluorescence values were converted to ΔF/F_0_, where F_0_ is the average fluorescence over the first 15 s of recording prior to addition of agonist (baseline) and ΔF is F_max_−F_0_. **(B)** The responses in A were quantitated by measuring the AUC and are presented relative to the AUC for 5 μM NMDA (100%). **(C)** Hippocampal cultures were preincubated with tPA (40 μg/ml) for 5 min or receptor-associated protein (RAP) (500 nM; for 15 min) and tPA (for 5 min). Baseline Fluo-4 fluorescence was monitored for 15 s prior to the addition of NMDA (5 μM), at time = 0. Fluo-4 fluorescence was monitored for a further 45 s. Raw fluorescence values were converted to ΔF/F_0_, where F_0_ is the average fluorescence over the first 15 s of recording prior to addition of agonist (baseline) and ΔF is F_max_−F_0_. **(D)** The responses in C were quantitated by measuring the AUC and are presented relative to the AUC for 5 μM NMDA (100%). RFU, Relative Fluorescent Units; n.s, not significant; ***p < 0.001. Error bar, SEM.

To investigate if tPA’s effects on intracellular calcium levels involved tPA-mediated activation of plasmin, cultures were preincubated with α_2_-antiplasmin. α_2_-antiplasmin (140 nM) alone did not affect 5 μM NMDA-induced calcium flux and did not block the effect on tPA on 5 μM NMDA-induced calcium flux (Figures 5A,B). This suggests that plasmin was not responsible for the observed tPA response. It does not rule out the possibility that tPA may be converting plasminogen to plasmin in our cultures, only that this conversion is not necessary for the observed effect of tPA on 5 μM NMDA-induced changes in calcium levels. As tPA has also been proposed to modulate NMDA-mediated calcium flux through interaction with LRP as a complex with a specific tPA inhibitor (Martin et al., 2008; Samson et al., 2008a) experiments were undertaken with the competitive LRP-1 receptor antagonist RAP. RAP alone had no effect on NMDA-mediated calcium flux (Figures 5C,D). RAP also failed to block the inhibitory effect of tPA on NMDA-mediated calcium flux (Figures 5C,D) suggesting that the tPA-mediated inhibition of 5 μM NMDA-induced calcium flux does not involve an interaction with LRP-1 or a similar RAP-sensitive receptor.

## Discussion

In this study we have explored the effect of the proteolytic enzyme tPA on NMDA receptor-induced calcium flux in primary cultures of rat hippocampal neurons. Our results reveal that tPA’s effects on NMDA-mediated changes in intracellular calcium levels vary with the concentration of NMDA. They support an inhibitory effect of tPA on calcium flux activated by low concentrations of NMDA or synaptic action potentials, through a mechanism that requires the enzymatic activity of tPA.

We found that pre-treatment of cultured hippocampal neurons with tPA had no effect on intracellular free calcium levels following stimulation with 50 μM NMDA. This relatively high concentration of NMDA has been reported to directly activate post-synaptic NMDARs (Bading et al., 1993; Hardingham et al., 2002). Our own results support this view with NMDA-mediated calcium flux activated by 50 μM NMDA inhibited by NMDA receptor antagonists but not by an antagonist of voltage-gated calcium channels. In contrast, tPA pre-treatment inhibited NMDA-stimulated calcium flux following activation of cultured hippocampal neurons with 5 μM NMDA. While all NMDARs are expected to be activated by this lower concentration of NMDA, the majority of calcium influx in hippocampal neuronal cultures has been attributed to an NMDA-induced increase in neuronal firing. Such action potential-induced intracellular calcium influx is mediated mainly by synaptic NMDARs (Hardingham et al., 2001a, 2002; Soriano et al., 2006). Our results support this view with significant inhibition of the calcium response following antagonism of L-type voltage-gated calcium channels, which have an established role in synaptically-stimulated calcium entry (Jensen and Wang, 1996; Wang and Gruenstein, 1997). We further investigated a role for tPA at synaptic NMDARs by examining its effect on synaptically-evoked bursts of action potentials. Hippocampal cultures typically contain approximately 10% inhibitory interneurons that tonically inhibit the neuronal network. We treated cultures with the GABA_A_ receptor antagonist bicuculline to relieve this inhibition, leading to bursts of action potentials and activity-dependent calcium transients mediated largely by calcium influx through synaptic NMDA receptor (Hardingham et al., 2001b). Co-treatment with 4-AP, a weak potassium-channel blocker led to elevated calcium oscillations with “plateau-type” calcium signal (Hardingham et al., 2002). Pretreatment of these cultures with tPA resulted in a marked decrease in calcium oscillations, further supporting an inhibitory effect of tPA on synaptic NMDARs.

To the best of our knowledge this is the first study to report an inhibitory effect of tPA on NMDA receptor-mediated calcium flux in neurons. Several other groups have investigated the effect of tPA on intracellular calcium levels (Nicole et al., 2001; Reddrop et al., 2005; Samson et al., 2008b). However in contrast to our results, these studies reported that tPA potentiated NMDA-mediated calcium levels. While these studies all involved analyses of mouse cortical cultures, either as a mixed population of neurons and glial cells (Nicole et al., 2001) or enriched in neuronal cells (Reddrop et al., 2005; Samson et al., 2008b), our inhibitory responses were seen in rat hippocampal cultures cultured under conditions to enrich for neuronal cells. Glial cells may influence tPA-mediated responses with tPA recently been described as a gliotransmitter (Cassé et al., 2012). Another obvious difference is the concentration of NMDA used across the different experiments. The tPA inhibitory effects we report were seen using 5 μM NMDA while the published studies used concentrations ranging between 25 μM and 100 μM. The higher concentrations of NMDA would be expected to result in cellular responses dominated by direct stimulation of NMDARs whereas the inhibitory effects we observe appear to be due to trans-synaptic stimulation of synaptic NMDARs. However, this suggestion alone does not explain why we do not see a potentiation of NMDA-mediated calcium levels at the higher NMDA concentrations. The differential effects of tPA on NMDA-mediated calcium flux could also involve differences in NMDA receptor subunit composition and differences in NMDAR responses reflecting association with regulatory proteins.

Our data support tPA inhibition of NMDA-mediated changes in calcium levels through a proteolytic mechanism. The changes could involve direct effects of tPA on the NMDA receptor to inhibit calcium entry but we cannot exclude changes in intracellular calcium also involving differential release of calcium from intracellular stores. Treatment of cultures with a catalytically inactive tPA mutant did not inhibit NMDA-mediated calcium flux. tPA has been proposed to cleave the GluN1 subunit of the NMDA receptor and potentiate NMDA-induced calcium influx (Nicole et al., 2001; Fernández-Monreal et al., 2004a; Reddrop et al., 2005; Benchenane et al., 2007). However, as all NMDARs contain the GluN1 subunit and as cleavage of this subunit potentiates NMDA-activated levels of intracellular calcium in neurons, it seems unlikely that the effects we are seeing are mediated through this subunit. Others have failed to detect cleavage of the GluN1 subunit of the NMDA receptor by tPA (Matys and Strickland, 2003; Kvajo et al., 2004; Liu et al., 2004). There is also evidence that plasmin can modulate NMDA receptor function directly (Samson et al., 2008a) or indirectly (Mannaioni et al., 2008). If plasmin were the functional unit in our assay conditions we would expect to see relief of the tPA-mediated inhibition of intracellular calcium levels when cultures were pre-incubated with the plasmin-specific inhibitor α_2_-antiplasmin. While, no such change was observed (Figures 4A,B), these experiments do not completely exclude a direct role for plasmin. Future experiments should investigate the effects of exogenous plasmin on NMDA-mediated calcium levels.

tPA has also been shown to interact with the NMDA receptor via the low-density LRP1. Several studies have implicated LRP1 as the major tPA receptor in the brain facilitation subsequent downstream signaling (Zhuo et al., 2000) and engagement of the LDLR has been reported to be required for tPA to influence NMDAR function, including potentiation of NMDA-mediated calcium influx (Samson et al., 2008a). In these studies the calcium responses of primary cortical cultures to 25 μM NMDA were measured before and after a 5 min perfusion with tPA by video microscopy. tPA was found to enhance NMDA-mediated calcium influx and the effect was blocked by the LDLR pan-ligand blocker RAP. Rather than this response reflecting direct binding of tPA to the LRP receptor, the authors suggest a model where tPA initially interacts with a substrate, which they suggest is protease nexin-1 (PN-1), and a member of the serine protease inhibitor family, and it is this complex that interacts with the LDLR to activate NMDA signaling. We saw no significant effect of RAP alone on NMDA-mediated changes in calcium levels. Moreover, in our assay conditions, RAP did not block tPA’s inhibitory effect. This may suggest that LRP’s main influence on NMDAR function, as previously reported, is through an interaction with extrasynaptic NMDARs.

Another potential target of tPA is GluN2D-containing NMDARs. tPA has been proposed to potentiate GluN2D-containing NMDA receptor-dependent activation in cortical neurons activated with 50 μM NMDA with receptor activation monitored by quantitation of ERK signaling or increased neuronal death (Baron et al., 2010; Jullienne et al., 2011; Parcq et al., 2012). Interestingly, tPA was unable to potentiate NMDA-mediated cell death in hippocampal cultures in this study, even though NR2D expression is seen in the CA2 region of the hippocampus. Investigations of tPA’s effect on NMDA-mediated changes in intracellular calcium levels in cortical cultures should shed further light on the role of GluN2D-containing NMDARs and is an important area for future study. Another consideration is the molecular form of tPA. tPA is secreted as single-chain tPA but can then undergo cleavage into a two-chain form by plasmin or kallikrein (Rijken et al., 1982; Rajapakse et al., 2005). Parcq et al. (2012) recently reported that single-chain tPA selectively cleaves the NMDA receptor to promote NMDA-induced calcium influx in mouse embryonic cortical cultures with two-chain tPA having no effect. Our experiments used human recombinant tPA (Actilyse) which is 90–95% single-chain tPA and a recombinant human tPA supplied by Biopur that is also the single-chain form. They further support an important role for single-chain tPA as a modulator of NMDAR responses. Whether two-chain tPA is inactive in our model remains to be determined.

In conclusion, our study has found that tPA can inhibit NMDA receptor-mediated changes in intracellular calcium levels in cultured primary rat embryonic hippocampal neurons when NMDARs are activated with either low concentrations of NMDA or through activation of synaptic NMDARs by blocking GABA_A_ receptor function. These effects require tPA to be proteolytically active and appear not to involve plasminogen as a substrate or LRP as part of a receptor-mediated mechanism. Our data provide additional evidence for the involvement of tPA in modulating NMDA receptor function and suggest a further level of complexity to the way that tPA may influence neuronal physiology and pathology. Further research is needed to determine which NMDA receptor subtypes are affected and how the effects relate to hippocampal synaptic plasticity *in vivo* (Liu et al., 2004).

## Conflict of Interest Statement

The authors declare that the research was conducted in the absence of any commercial or financial relationships that could be construed as a potential conflict of interest.
