# Supplementary figures and images for: Tissue plasminogen activator inhibits NMDA-receptor-mediated increases in calcium levels in cultured hippocampal neurons
Source: Front Cell Neurosci. 2015 Oct 9;9:404. doi: 10.3389/fncel.2015.00404 (PMC4598481; doi:10.3389/fncel.2015.00404)

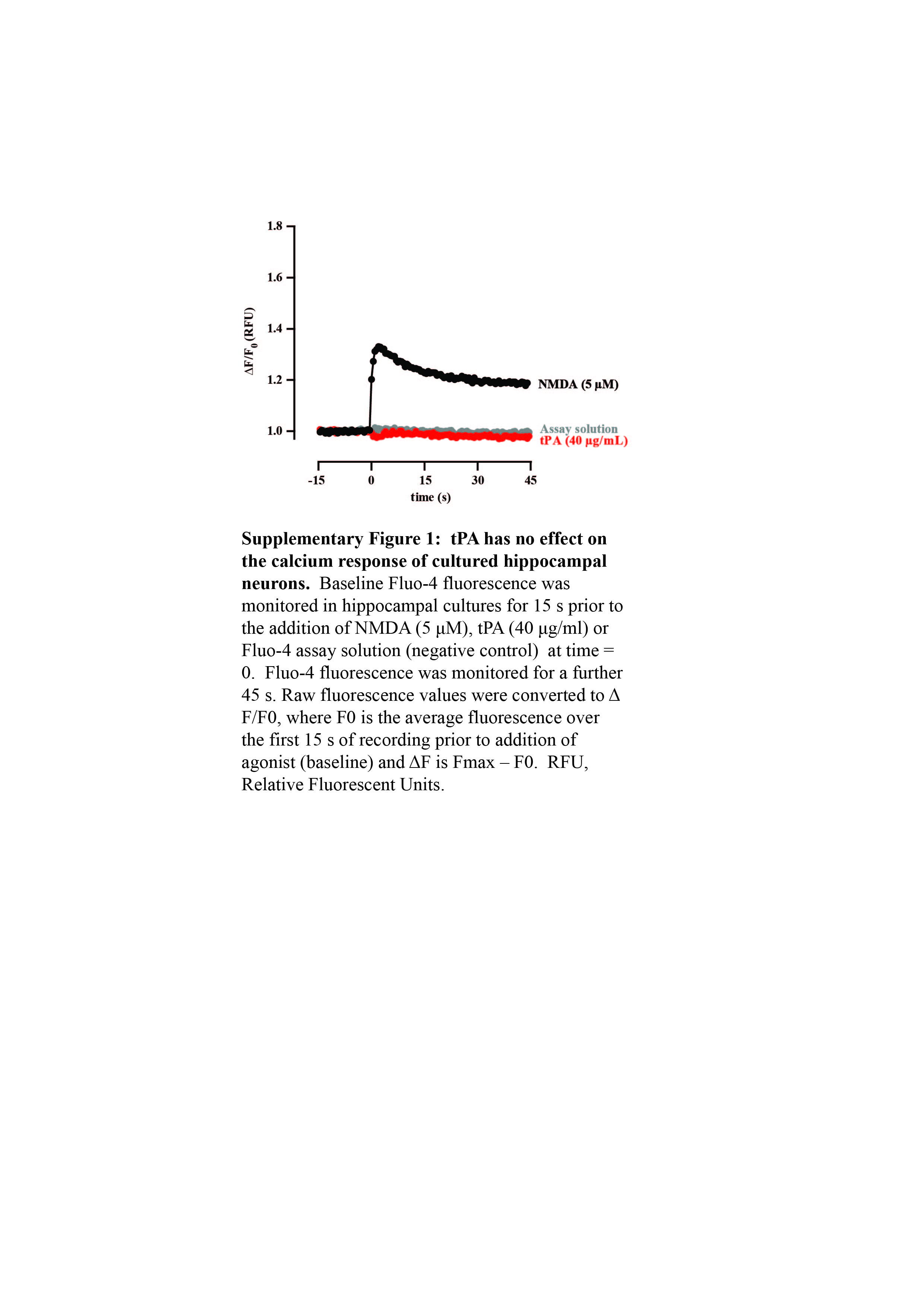

Supplement: Supplementary file 1 [file Image_1.JPEG]

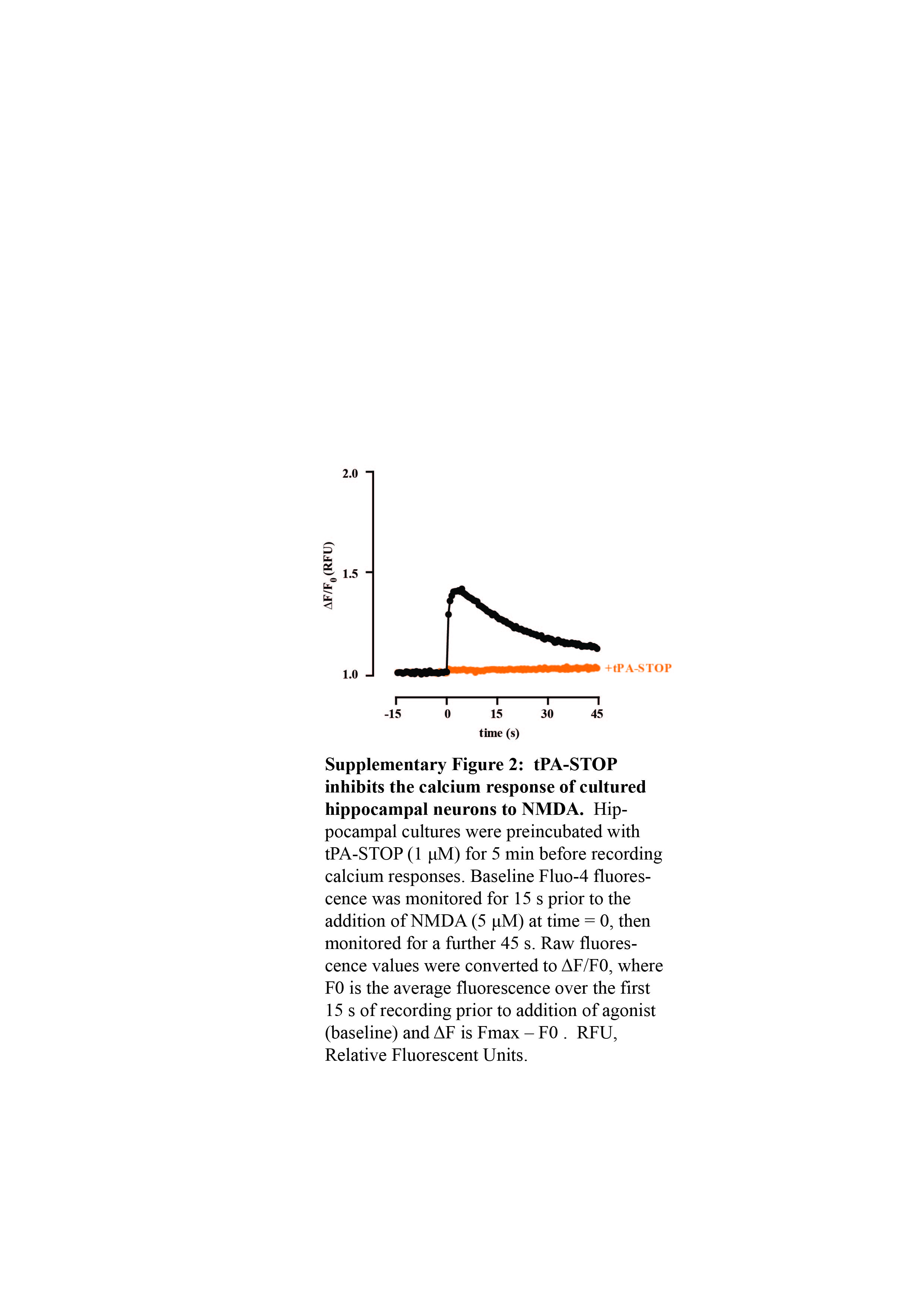

Supplement: Supplementary file 2 [file Image_2.JPEG]
